# Preclinical septic shock research: why we need an animal ICU

**DOI:** 10.1186/s13613-019-0543-6

**Published:** 2019-06-10

**Authors:** Antoine Guillon, Sebastien Preau, Jérôme Aboab, Eric Azabou, Boris Jung, Stein Silva, Julien Textoris, Fabrice Uhel, Dominique Vodovar, Lara Zafrani, Nicolas de Prost, Peter Radermacher, Translational Research Committee of the French Intensive Care Society (Société de Réanimation de Langue Française)

**Affiliations:** 1 Service de Médecine Intensive - Réanimation, CHRU de Tours, Tours, France; 2 Centre d’Etude des Pathologies Respiratoires (CEPR), UMR 1100, INSERM, Faculté de Médecine, Université de Tours, Tours, France; 3 Service de Médecine Intensive, Hôpital Salengro, CHU Lille, Lille, France; 4 Lille Inflammation Research International Center (LIRIC), U 995, School of Medicine, INSERM, Univ. Lille, Lille, France; 5 Service de Réanimation, Hôpital Delafontaine, Saint-Denis, France; 6 Service de Réanimation, Assistance Publique-Hôpitaux de Paris, Hôpital Raymond Poincaré, 92380 Garches, France; 7 Service de Réanimation, CHU de Montpellier, Montpellier, France; 8 Service de Réanimation, CHU Purpan, 31300 Toulouse, France; 9 Département d’Anesthésie-Réanimation, hôpital Édouard-Herriot, Hospices Civils de Lyon, CHU de Lyon, 69437 Lyon, France; 10 EA 7426 Pathophysiology of Injury-induced Immunosuppression, University of Lyon1-Hospices Civils de Lyon - bioMérieux, Hôpital Edouard Herriot, 69437 Lyon, France; 11 Service de Réanimation Médicale et Maladies Infectieuses, CHU de Rennes, Hôpital Pontchaillou, Rennes, France; 12 Centre Antipoison et de Toxicovigilance de Paris – Fédération de Toxicologie, Hôpital Fernand-Widal, Assistance Publique-Hôpitaux de Paris, Paris, France; 13 UMRS 1144, Faculté de Pharmacie, INSERM, Paris, France; 14 Service de Réanimation Médicale, Assistance Publique-Hôpitaux de Paris, Hôpital Saint-Louis, Paris, France; 15 Service de Réanimation Médicale, Hôpital Henri Mondor, Assistance Publique-Hôpitaux de Paris, 51, Avenue du Maréchal de Lattre de Tassigny, 94010 Créteil Cedex, France; 16 Institut für Anästhesiologische Pathophysiologie und Verfahrensentwicklung, Universitätsklinikum, Ulm, Germany

**Keywords:** Animal models, Septic shock

## Abstract

Animal experiments are widely used in preclinical medical research with the goal of disease modeling and exploration of novel therapeutic approaches. In the context of sepsis and septic shock, the translation into clinical practice has been disappointing. Classical animal models of septic shock usually involve one-sex-one-age animal models, mostly in mice or rats, contrasting with the heterogeneous population of septic shock patients. Many other factors limit the reliability of preclinical models and may contribute to preclinical research failure in critical care, including the host specificity of several pathogens, the fact that laboratory animals are raised in pathogen-free facilities and that organ support techniques are either absent or minimal. Advanced animal models have been developed with the aim of improving the clinical translatability of experimental findings. So-called animal ICUs refer to the preclinical investigation of adult or even aged animals of either sex, using—in case of rats and mice—miniaturized equipment allowing for reproducing an ICU environment at a small animal scale and integrating chronic comorbidities to more closely reflect the clinical conditions studied. Strength and limitations of preclinical animal models designed to decipher the mechanisms involved in septic cardiomyopathy are discussed. This article reviews the current status and the challenges of setting up an animal ICU.

## Introduction

Animal experiments are widely used in preclinical medical research for modeling disease and for exploring novel therapeutic approaches. In the context of sepsis and septic shock, the translation into clinical practice has been fairly disappointing [1]. Clearly, models are to mirror key features of the condition to be studied, but, nevertheless, they are not capable of completely replicating all aspects due to inherent simplification. This issue was elegantly summarized by the British statistician George Box: “*Essentially, all models are wrong, but some are useful*” [2]. Certainly, in the context of sepsis and septic shock, the lacking translation of animal experiments to clinical practice is at least in part due to the complexity of the disease state per se. Moreover, the discrepancy between promising experimental findings and disappointing translation into the clinical setting has also been referred to the inappropriateness of the models used, which ultimately often results in “*experimentally induced disturbances that usually diverge from naturally occurring human illness, precluding the replication of disease dynamics and time course*” [3]. Moreover, the authors of the latter review stated that *“experiments with more advanced supportive care[…]*, *permitting the testing of drugs in a more realistic setting* “ should be applied [1]. It is self-evident that this raises the question whether an “animal ICU” is necessary. An “animal ICU” refers to investigation of adult or even aged animals of either sex, eventually with underlying chronic comorbidities and with—in the case of rodents—miniaturized equipment allowing for reproducing an ICU environment, which would ideally integrate standard interventions (including antibiotics, fluids, monitoring) used in the clinical setting of sepsis (see below for details). This discussion is by no means new: Two decades ago, Daniel Traber already highlighted this problem: “*Would you as a critical care physician accept data on a septic patient who was not resuscitated? Would you accept data from a drug study on an intensive care patient who was not only not resuscitated with fluid but who did not even have blood pressures and heart rates monitored? If the animals are resuscitated, is the resuscitation to a specific physiologic variable? The pathophysiology and outcome of an unresuscitated, unmonitored, septic patient is certainly different*” [4]. The objectives of the present article are (1) to discuss the strengths and limitations of the currently used animal models of sepsis/septic shock, (2) to review the current status and the challenges of setting up an animal ICU and (3) to highlight the relevance of an ICU animal model in the specific example of the septic cardiomyopathy. In this context, this article is complementary to the most recent publications on the “*Minimum quality threshold in pre*-*clinical sepsis studies (MQTIPSS)*” initiative of the Wiggers Bernard Conference in Vienna, May 2017 [5–8].

## Classical animal models of sepsis/septic shock: strengths and limitations

### Do not abandon yet the mouse research ship

**Fig. 1:**
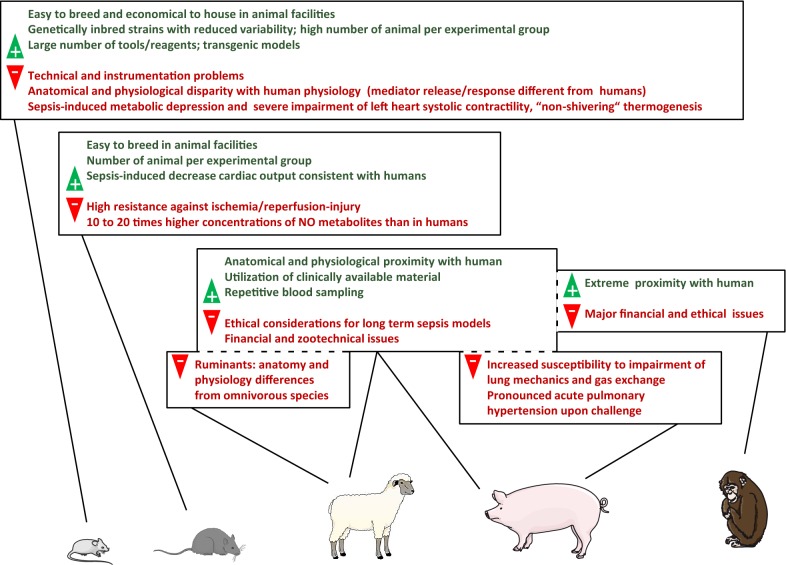
“Pros” and “Cons” of experimental models for septic shock research

Animal models allow for testing hypotheses generated in patients using intact living systems, so that currently there is no better way to bridge between patients and the laboratory bench. When setting up an animal model, the question that immediately arises is: “Which species should be studied?” Clearly, the most frequently used species is the mouse because of the availability/price of the animals, the accessibility of specific reagents and the development of genetically modified mice. The “pros” and “cons” of the most frequently used mammal species are summarized in Fig. 1. Basic laboratory observations, mechanistic studies or pre-treatment assessment can be performed either by blocking or stimulating key pathways with specific effectors and/or by genetic manipulation, ultimately allowing sophisticated analysis. Gene editing permitted by the CRISPR-Cas9 system now gives the ability to change an organism’s DNA and has recently generated a lot of excitement in the scientific community [9]. There are obvious anatomical and physiological limitations while extrapolating results from a 20–30 g rodent to a 70–80 kg human adult. However, one has to remember that one of the most ground-breaking immunological discoveries was obtained in flies (*Drosophila melanogaster)*: Toll pathway cascade and the subsequent characterization of toll-like receptors have reshaped our understanding of the immune system [10]. Indeed, it is important to keep in mind that even the most simplistic models, in particular mouse models, are potent experimental models for biological questions and/or proof-of-concept studies. A vast amount of researches over the last decades has resulted in the development of numerous examples of valid mouse-to-human translation [11]. These models are the key resource for many biological explorations. The problem of experimental variability can be easily overcome while studying small animals by increasing the sample size, whereas it can hardly be in larger species due to technical and financial concerns. A number of excellent reviews have highlighted interspecies anatomical and physiological differences to help in the selection of appropriate animal models and ensure successful transposition in the clinics [12–16]. Indeed, a major strength of mouse models is to be able to promptly test scientific hypothesis in proof-of-concept studies, whereas the investments in personnel, equipment and consumables to develop advanced animal models can be prohibitive in early stage projects. Indeed, both models (“regular” mice models and advanced animal models) have their own advantages and limitations, but their aims are different and both are needed.

### Why mouse models may poorly mimic human responses

Anatomical and physiological differences are probably the visible tip of the iceberg in terms of barriers to extrapolation from experimental models. Some other gaps are less obvious. First, age- and sex-matched animal models are carefully designed to standardize the confounding factors and to minimize heterogeneity of the results. Indeed, conclusions of experimental research are generally drawn from one-sex-one-age animal models (generally of young age to reduce the cost). In contrast, sepsis affects individuals of all ages, size/weights and sex. This “one size fits all” strategy is understandable in order to reduce animal consumption as recommended by the “3R” principles (“all efforts to replace, reduce, and refine experiments must be undertaken” [17]), but transposing the conclusions to a highly heterogeneous human population is questionable. Similarly, preclinical models cannot reproduce the full complexity of the clinically preexisting health conditions.

**Fig. 2:**
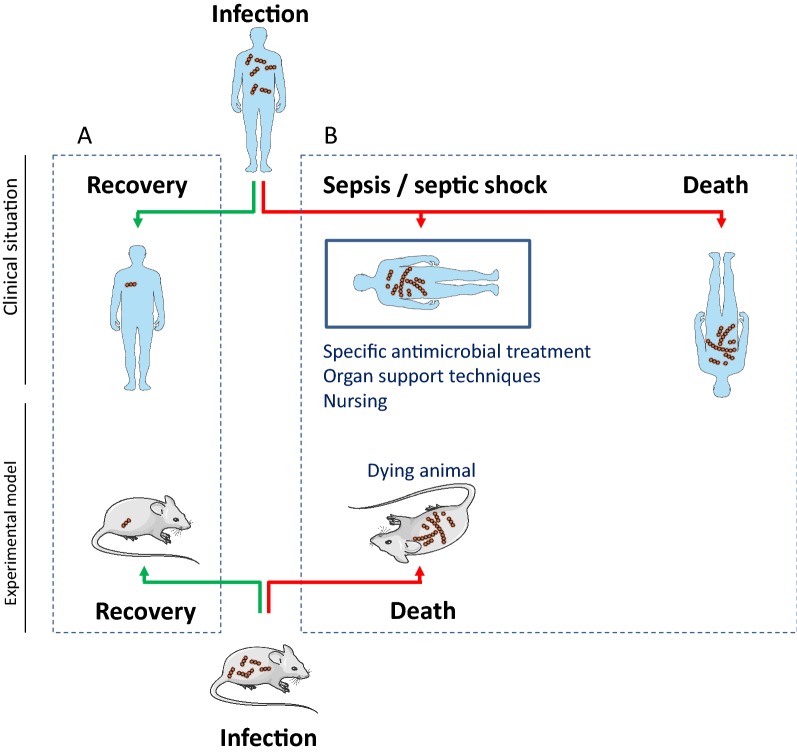
Limits of classical animal models regarding sepsis/septic shock. **a** Infected animals are powerful models for basic laboratory observations and/or mechanistic studies of host/pathogen interactions. **b** Animals with a severe infection have two options, curing or dying, while human has an “intermediate” possibility, surviving. ICU patients represent an abnormal situation to an evolutionary view that is difficult to replicate. Infected animals with lethal dose of microbial agents are killed at a defined time point and compared with septic ICU patients. The ICU environment (monitoring, nursing) and treatments (specific antimicrobial treatment, organ support technics) are rarely reproduced in the animal models leading to an uncontrolled mismatch between animal models and “real life” of human care

Second, regarding sepsis and host–pathogens interaction, a major problem is the host specificity of most pathogens [18–20]. As an example, mice are not susceptible to infection by human influenza A virus strains. Some of the virus strains must be adapted to be virulent in mice by serial passaging [21]. Similarly, our current understanding of immunology is largely defined in laboratory mice. However, there is growing concern that laboratory mice do not reflect relevant aspects of the human immune system, as laboratory mice have a less complex immune system and live in abnormally hygienic specific pathogen-free barrier facilities [22, 23]. Humans are infected with a variety of acute and chronic pathogens over the course of their lives, and pathogen-driven selection has shaped the immune system of humans. The same is likely true for mice. However, laboratory mice we use for most biomedical studies are bred in ultra-hygienic environments and are kept free of specific pathogens [24]. The immune system of a human adult is probably more closely replicated by mice caught in the wild or purchased in pet stores [22]. Indeed, mice with diverse environmental exposures have more mature immune responses, similar to human adults, whereas laboratory mice have immature or neonate-like immune systems. Moreover, in mouse models the genomic response to endotoxemia, trauma and burns was reported to only poorly mimic that of healthy volunteers and/or patients [25]. It is noteworthy in this context, however, that using the same database other authors came to exactly the opposite conclusion, i.e., that “*mouse models greatly mimic human inflammatory diseases*” [26]. Third, an animal with a severe infection has two options: rapidly succumb to sepsis or spontaneously recover. A human being with a severe infection has an “intermediate” possibility: surviving (i.e., prolonged critical illness permitted by health care). Severely ill patients are placed in an unphysiological status due to organ support techniques, specific drugs or, in more general terms, all treatments provided in the ICU. There is not a single mechanism at play, in contrast to the initially responsible disease, but a high variety of interplaying mechanisms including effects of all the therapeutic strategies involved. The spectrum of causes or effects is finally difficult to decipher. Because of the level of complexity, animal models incompletely replicate ICU patients. Septic ICU patients are compared with animals infected with lethal dose of microbial agents and killed at a time point defined by the expected survival curve (Fig. 2). Neither the long time course of the human disease nor the complex ICU environment/treatments could be accurately put into the equation.

## Advanced animal models comprising full-scale ICU treatment

### Do we need the development of miniaturized and sophisticated equipment to study resuscitated rodent models?

**Fig. 3:**
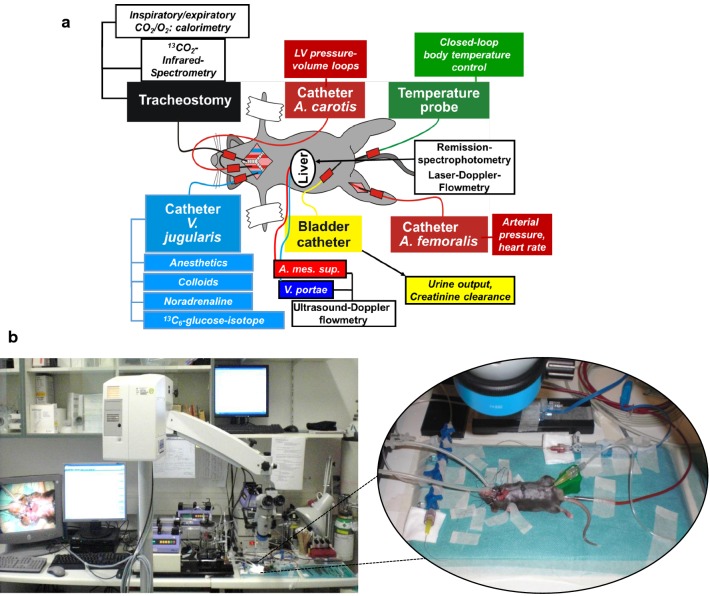
ICU environment in murine experiments. **a** Schematic representation of the monitored parameters; **b** representative illustration of miniaturization of sophisticated equipment used to reproduce the ICU environment in murine experiments; A. carotis, artery carotis; A. mes. sup., artery mesenteric superior; v. portae, vein portae; A. femoralis, artery femoralis

As mentioned above, Angus and van der Poll [1] suggested that experiments with more advanced supportive care would permit the testing of drugs in a more realistic setting. For models of sepsis and septic shock, this would ideally require providing appropriate antimicrobial treatment, hemodynamic monitoring together with adequate fluid resuscitation as well as—if necessary—vasopressor/inotropic support, and eventually (“lung-protective”) mechanical ventilation. Such a strategy of using resuscitated animals indeed increases the chance of successful translation of animal experiments to clinical practice: In fact, already in 2001, Hollenberg et al. demonstrated the impact of standard ICU treatment on mortality of polymicrobial septic shock. Combining fluid resuscitation with antibiotics allowed achieving a 46% survival at 48 h in a murine model of cecal ligation and puncture [27]. In other words, investigating resuscitated animals allowed for approximating mortality rates comparable to the clinical reality rather than studying an otherwise 100% lethal model. This approach also allowed to characterize the role of the inducible isoform of the nitric oxide synthase (iNOS). While 48-h survival did not significantly differ between wild type and iNOS knockout (iNOS^−/−^) in animals without ICU treatment (0 vs. 13%), iNOS^−/−^ mice showed a markedly improved outcome, as compared with wild-type animals, after administration of antibiotics plus fluid resuscitation (55 vs. 24%) [28]. Moreover, development and miniaturization of sophisticated equipment has allowed for overcoming many technical problems that hindered or even prevented the establishment of ICU procedures in murine experiments (Fig. 3). This is particularly true for mechanical ventilation, which was originally harmful per se due to the lacking availability of appropriate small animal ventilators and/or the use of injurious ventilator settings, in particular as a result of high tidal volumes and/or inadequate positive end-expiratory pressure (PEEP) levels. In contrast, the recently developed small animal mechanical ventilators specifically designed for mice and/or rats allow for implementing “lung-protective” mechanical ventilation with the use of low tidal volumes (6–8 μL/g BW [29], adjustment of PEEP and maximum airway pressures according to gas exchange measurements, thereby following the guidelines of the ARDS Network and/or using the lower and upper inflection points obtained from the determination of pressure–volume curves [30], and inflation hold maneuvers to recruit atelectatic lung regions [31].

### Do we need to study animal models in larger species?

Despite the above-mentioned technical advances, mice as experimental animals present with a major pitfall for translational research, namely due to their fundamentally different metabolic response to stress states resulting from their ability to rapidly reducing “*non*-*shivering thermogenesis*” and thereby core temperature [32]. Hence, hypothermia (if not corrected by external means) is a characteristic feature of the murine adaptive response to stress, whereas in larger species as well as in humans it is a mirror of whole body energetic failure (unless therapeutically induced). This problem can be overcome by replacing mice with rats, which, given the size of the individual animals, obviously also alleviates numerous technical and instrumentation problems. Indeed, during sepsis and septic shock, rats were demonstrated to much more closely resemble human beings than mice, in particular with respect to their long-term metabolic and cardiovascular profile [33]. Nevertheless, a word of caution should be left on the translational value of rat experiments, in particular in the context of circulatory shock: Nitric oxide (NO) is referred to as the “final mediator” of vasodilation and impaired vasoconstrictor response in septic shock, and the activity of the inducible isoform of the NO synthase (iNOS) is particularly pronounced in rats, which results in five- to tenfold higher nitrite and/or nitrate blood levels in septic and/or endotoxemic rats when compared to larger species (e.g., swine or sheep) and human beings [34–36]. Moreover, rats show pronounced antioxidant enzyme activity, which, together with the high NO release, makes them particularly resistant against ischemia/reperfusion (*I*/*R*) injury [37, 38].

Larger species, in particular when human-sized individual animals are studied, allow for utilizing clinically available material, which obviously facilitates anesthesia and surgical instrumentation, monitoring and the choice of interventional targets. Moreover, the large blood volume permits repetitive blood sampling and titration of readouts according to routinely available biomarkers as well as to physiological target endpoints, as in human. Sheep and swine are the most commonly used larger species, and various physiological parameters are even numerically comparable to the values found in age- and/or sex-matched patients. The “pros” and “cons” of this species are summarized in Fig. 1. As humans, they are monitored and/or vaccinated for specific pathogens and are exposed to an environment rich of microbial exposure. Nevertheless, despite the well-spread and the apparently close similarity of physiological and biological readouts, there are important species-specific differences to keep in mind. For example, sheep are ruminant animals and their gastrointestinal anatomy and physiology markedly differs from the omnivorous species swine, which translates into different kinetics of glucose metabolism. Swine rapidly develop profound acute pulmonary hypertension [39, 40] and are susceptible to impairment of lung mechanics and gas exchange [41, 42]. While in sheep, similar to human beings, measuring the creatinine clearance is an adequate marker of glomerular filtration rate (GFR) [43], this can result in overestimation of GFR in swine due to tubular creatinine secretion [44]. Currently, the mean duration of the ICU stays of patients for septic shock is one to several weeks. In contrast, the maximum duration of stay in an animal ICU is much shorter. Clearly, logistical constraints, in particular due to the required staff, assume major importance, but again, the choice of the species studied plays a major role, in particular with respect to the duration of mechanical ventilation (even without any additional challenge, e.g., circulatory shock and/or hyper-inflammatory conditions). The smaller the animal, the shorter the maximal duration of mechanical ventilation, even with the use of standard clinical ventilator settings to avoid injurious ventilation and, hence, to prevent or at least limit any ventilator-induced lung injury: Whereas the maximal duration of mechanical ventilation in mice and rats is 10–16 [45, 46] and 18–24 h [47, 48], respectively, larger species have been studied during much longer periods of mechanical ventilation, for even up to 48–96 h in sheep [49–52], 54 h in nonhuman primates [53] and 72–104 h in swine [54, 55]. Noteworthily, the limitation concerning one-sex-one-age in mice models (in contrast to the heterogeneous population of septic shock patients) still exists while using larger species.

### Do we need to study animals with various age, sex and underlying comorbidities?

It is well established that age, sex and, in particular, chronic comorbidities markedly influence the outcome after sepsis and/or circulatory shock. This notion is also true for preclinical animal experiments, for which the importance of age, sex [56, 57] and/or underlying chronic diseases, including chronic obstructive pulmonary disease (COPD) [30, 58, 59] or cystic fibrosis [60], atherosclerosis [61], cancer [62], diabetes [63, 64] or even “psychological stress” [65], has been demonstrated. Indeed, preexisting coronary artery disease even completely abolished [66, 67] the otherwise promising results obtained in comparable experimental settings in young and healthy animals [68, 69]. Nevertheless, so far, the vast majority of experiments using an animal ICU, in particular when rodents are studied, investigate(d) young and healthy individuals. Clearly, it must be emphasized that since any added comorbidity will aggravate the burden to the animal, integrating comorbidity into the experimental design will necessarily create an ethical dilemma: Is it better to avoid comorbidities to reduce this burden but likely generate less relevant data, or include them expecting that a given comorbid setting might produce novel and translatable findings? [5, 62]. This dilemma obviously is also true for the use of mechanical ventilation, which per se may induce hemodynamic compromise and/or additional lung injury, but most likely will also allow for deeper anesthesia and sedation and thereby alleviate respiratory distress.

## Contribution of animal experiments in our current understanding of sepsis: the example of septic cardiomyopathy

Although a precise definition of septic cardiomyopathy is still a matter of debate, cardiac dysfunction occurs in 40–60% of septic shock patients. It is entirely reversible in survivors within 10 days [70], but the presence of sepsis-related cardiac dysfunction is associated with increased short-term mortality [71–74]. Apart from causal treatments (antibiotics, surgical source control) and initial fluid resuscitation, no available treatment has been shown to improve the course of septic cardiomyopathy in humans [75]. As myocardial tissue is not easy to obtain from critically ill patients, only a few studies displayed data on the cellular mechanisms of human septic cardiomyopathy from the left ventricle of patients who died from sepsis [76–79]. To guide the understanding of the main causal mechanisms of human septic cardiomyopathy, most animal studies have used either endotoxin or fecal peritonitis models, with no or only limited resuscitation. Cecal ligation and puncture (CLP) models mimic the chronology of morphological features observed in human septic cardiomyopathy. Early in this model, prior to fluid resuscitation, animals show decreased cardiac output [80] with normal cardiac contractile performances of isolated perfused hearts [81]. At this stage, fluid resuscitation normalizes cardiac output, leading to the so-called hyperdynamic phase of sepsis [82]. Later on, animals that are more likely to die display decreased cardiac output [80] due to cardiac contractile dysfunction (i.e., the “hypodynamic phase of sepsis”) [81]. Conversely to the CLP model, endotoxin models (e.g., using bacterial lipopolysaccharide (LPS)) show very early cardiac contractile dysfunction, skipping the phase of hemodynamic disturbances with maintained cardiac performances [83, 84]. Surviving animals experience total recovery of their cardiac performances within 10 days [85–87]. Unlike endotoxin models, infected animals that mimic the different phases of human septic cardiomyopathy were very useful to analyze the early pathophysiological mechanisms of this syndrome. For example, CLP animals display an early increase in the adrenergic response of the cardiomyocytes, altering calcium sensitivity of cardiac myofibrillar proteins [81, 88–90]. This early mechanism is at least in part responsible for the later attenuation of adrenergic response and septic cardiomyopathy [81, 88–90]. Thus, these CLP-specific data helped researchers to hypothesize that preventing adrenergic stimulation in the early phase of sepsis might prevent cardiomyopathy to occur. Although CLP models are usually fluid-resuscitated, only a minority of the studies used antibiotic regimens [80, 82], and none of them performed complete causal treatments (i.e., both antibiotics administration and surgical source control). Therefore, one might hypothesize that like CLP models with only partial basic resuscitation, CLP models with full causal treatment could bring new significant insights on the pathophysiological mechanisms of septic cardiomyopathy (i.e., still “wrong” but maybe more “useful” models [2]).

Finally, preclinical trials on septic cardiomyopathy yielded conflicting results with poor extrapolation to critically ill patients. Several factors might explain the failure to detect new efficient drugs in this context. First, numerous preclinical experiments were performed on endotoxin models, known to poorly mimic human features of the septic cardiomyopathy [91–100]. Second, like mechanistic studies, although preclinical trials using fecal peritonitis models were usually fluid-resuscitated, none of them combined a causal treatments to the tested drugs [91, 101–108]. Third, only a few studies administered vasoconstrictors to maintain an appropriate systemic blood pressure when testing heart medications with vasodilator properties [107, 108]. Fourth, tested drugs were sometimes administered very early (< H4) [105, 106] after the induction of fecal peritonitis, or even before the insult [91, 92, 109]. All these factors might have favored the beneficial effects of the tested drugs and led to false positive results. Therefore, although sophisticated treatments (e.g., mechanical ventilation) may improve the quality of animal studies in the context of septic cardiomyopathy [110], implementation of the most basic causal treatments should be a prerequisite for future preclinical studies in this field.

## Conclusion

Preclinical models have been widely used with the ultimate goals of improving the underlying mechanisms of the disease and exploring new therapeutic approaches. Simplistic models, in particular mouse models, are potent experimental models for biological questions and/or proof-of-concept studies but failed to bridge the translational gap to the clinic in the setting of septic shock. Using advanced animal models, namely integrating the investigation of adult (and/or aged) animals of either sex in the presence/absence of underlying chronic comorbidities under standardized animal ICU environments, i.e., by integrating as much as possible the standard interventions (including antibiotics, fluids, monitoring) used in the clinical setting of sepsis, will help to overcome the classical limitations of previous experimental studies performed on septic shock and enhance their translational value.

## Data Availability

Not applicable.

## Abbreviations

CLP: cecal ligation and puncture
ICU: intensive care unit
